# “It doesn’t matter if we’re the most amazing professionals in the world…” A qualitative study of professionals’ perspectives on parent-child interaction assessment with deaf infants

**DOI:** 10.3389/fpsyg.2024.1315220

**Published:** 2024-03-04

**Authors:** Martina Curtin, Tina Wakefield, Rosalind Herman, Gary Morgan, Madeline Cruice

**Affiliations:** ^1^Speech and Language Therapy, Homerton Healthcare NHS Foundation Trust, London, United Kingdom; ^2^Language and Communication Science, City, University of London, London, United Kingdom; ^3^National Deaf Children’s Society, London, United Kingdom; ^4^Universitat Oberta de Catalunya, Psychology and Education Sciences, Barcelona, Spain

**Keywords:** parent-child interaction, deaf, assessment, professional practice, focus groups, infant, caregiver

## Abstract

**Introduction:**

Parent child interaction (PCI) is positively associated with deaf children’s language development. However, there are no known, deaf-specific tools to observe how a parent interacts with their deaf child aged 0–3 years. Without a framework for professionals to use with families, it is unknown how professionals assess PCI, what they assess, why they assess, and how the assessment results relate to case management.

**Methods:**

Eighteen hearing and deaf professionals, who work with deaf and hard of hearing infants aged 0–3 years and their families, attended online focus groups. The aim of the study was to gain insight into the professional assessment of PCI. Data were analyzed using thematic analysis.

**Findings:**

Six themes were generated from the dataset. Professionals discussed how central parents were in the support offered to families in the home, the importance of knowing and understanding the individual family, and accounting for and supporting parental wellbeing. Descriptions on how to administer a best practice PCI assessment included which parent behaviors to assess and how to make adaptations for different populations. Professionals shared how the assessment and review process could be used to inform and upskill parents through video reflection and goal setting.

**Discussion:**

This study provides insight into the mechanisms and motivations for professionals assessing the interactive behaviors of parents who have deaf children aged 0–3. Professionals acknowledged that family life is multi-faceted, and that support is most meaningful to families when professionals worked with these differences and incorporated them into assessment, goal setting, and intervention plans.

## Introduction

Deafness is a low incidence condition with estimated prevalence of 1 per 1000 live births (Morton and Nance, 2006). According to the most recent UK-wide summary from the Consortium for Research in Deaf Education (The Consortium for Research in Deaf Education [CRIDE], 2022), there are 52,798 deaf children and young people in the UK (aged 0–19 years). In this paper, we use the term ‘deaf’ to refer to all levels of deafness, from mild to profound. Although deafness is low incidence, it is a high need, long-term condition. Further, this population is highly diverse due to differences in levels of deafness, audiological equipment provision, age of fitting and levels of consistent use (hearing aids, implants, or none), language choices (monolingual spoken language users, multilingual spoken language users, sign language users, bimodal/bilingual language users), a high incidence of comorbid difficulties (40% according to Cejas et al., 2015), and other more universal differences such as maternal education, socio-economic status and levels of family involvement.

Most deaf children are born to hearing families (Mitchell and Karchmer, 2004) who have not yet developed effective skills in communicating with their deaf children. Reduced or disrupted input affects how a child develops language (Levine et al., 2016). Indeed, deaf and hard of hearing children’s language is reported to be 1–1.5 standard deviations lower than hearing peers (expressive and receptive spoken language in Ching and Dillon, 2013; expressive spoken language vocabulary in Yoshinaga-Itano et al., 2017). A recent systematic review found that parents’ linguistic input explained 31.7% of the variance in deaf children’s expressive language (Holzinger et al., 2020). Parents of deaf children therefore need to be supported to adapt their communication style to attain successful interactions (Dirks and Rieffe, 2019). Early interventions that coach parents to use supportive interaction strategies help to improve deaf children’s communication skills (Roberts, 2019; Nicastri et al., 2021). An important early step in providing targeted support and intervention is parent-child interaction (PCI) assessment. However, to date, a deaf-specific, validated tool of PCI does not exist.

A recent systematic review summarized 61 papers (Curtin et al., 2021) and identified which PCI behaviors are assessed in research with deaf children. These were: attention-getting, joint engagement, emotional availability, and responsivity of a parent and strategies for providing accessible and stimulating linguistic input. Most researchers focus on the mother-child dyad in PCI and these interactions are often filmed in labs, for 20 min on average. Researchers mostly used frame by frame analysis with coding systems. The review found that the length of joint engagement between parent and child, the level of parental sensitivity and the use of parental communication behaviors were significantly correlated with greater gains in deaf children’s language. Whilst it is beneficial to consider how PCI is assessed in research, the highly rigorous methods used are time consuming and unlikely to have application in real-life clinical settings. Professional practice therefore needs consideration.

In the United Kingdom, the first professionals to support families of deaf children at home are Qualified Teachers of Deaf Children and Young People (QToDs) and Speech and Language Therapists (SLTs). Tools such as the Ski-Hi Language Development Scale (Watkins, 2004), the MacArthur Communicative Development Inventory (Fenson et al., 1993), the Visual Communication and Sign Language (VCSL) Checklist for Signing Children (Simms et al., 2013), and Success from the Start (National Deaf Children’s Society, 2020) assist professionals with monitoring a deaf child’s communication development. Similarly, the Tait video analysis method (Tait et al., 2007) focuses on the deaf child’s eye gaze, and vocal and auditory pre-verbal skills, even though it is recommended to keep the adult’s face/profile within the camera’s shot. These tools observe and monitor one interactant and do not explicitly observe parents’ *interaction skills* when they are communicating with their deaf child. There are no known, deaf-specific assessment tools for observing parent interaction, despite this being a known predictor for language development. The lack of a reliable, evidence-based assessment tool may mean that professionals are not in agreement on which parental behaviors are important to appraise in the home, do not have a shared technical language when discussing assessment findings, and/or do not offer standardized care. This lack of consensus can increase the chances of disparity between professionals on how to identify parent and child strengths, needs, and areas to address in intervention. In turn, this can impinge on the child’s language development if therapy goals are not appropriate.

A survey of 190 UK-based professionals working with deaf 0–3-year-old children (Curtin et al., 2023) found that PCI was routinely assessed by the majority of professionals, and that there was substantial overlap between professional groups in which parent behaviors are assessed. Many professionals observed parent behaviors identified in Curtin et al. (2021). Survey participants (Curtin et al., 2023) reported an additional 18 novel parent behaviors they felt were missing from the survey, e.g., parent using appropriate voice volume, using a range of different word types, offering and labeling choices. Furthermore, professionals’ methods of assessment were informal and predominantly consisted of observation and note making. The vast majority of professionals used their own skills and experience to analyze interactions rather than any adapting any existing tools from the hearing population. Goal setting is a regular part of parent-implemented and/or parent-focused intervention (Barnett et al., 2023) and many of the professionals in the survey reported deciding upon goals with parents. What was not clear from the survey was why professionals assess PCI, how they introduce the concept to families, and how they work with or include aspects of everyday life that might impact a family’s interactions, such as a parents’ wellbeing or a deaf-plus child (i.e., a deaf child with additional needs). Finally, the survey did not explore goal setting practices in depth, and how these might differ across families.

In practice, considerable expertise and knowledge are required to observe and make sense of PCI. Despite professional bodies recommending that PCI be monitored (Royal College of Speech and Language Therapists [RCSLT], 2019), there is little evidence or guidance of how to do this in practice. The current study is third in a series of five that aim to develop an evidence-based assessment tool for PCI with deaf children aged 0–3 years. Combined with the earlier professionals’ survey (Curtin et al., 2023), this work seeks to gain insight into the motivations and mechanisms for the professional assessment of PCI (i.e., the why and the how). Specifically, it focuses on why PCI assessment is important, features of best practice, how to assess, what to assess, and how assessment relates to case management. Findings generated from the focus groups aim to enhance the knowledge and skills of professionals more widely.

## Research questions

1. Why is assessing parent behaviors in early PCI important, when the deaf infant is aged 0–3?
2. How do professionals conduct a best-practice PCI assessment when the infant is deaf aged 0–3?
3. Which parent behaviors are most important to assess?
4. How do PCI assessments influence professionals’ practice?

## Materials and methods

This study formed part of a large, explanatory, sequential mixed-methods project lead by the first author. First, data were collected via an open quantitative survey of 190 UK-based professionals (Curtin et al., 2023). The analysis of the survey guided the planning of the follow-up qualitative focus groups. In this paper, we report the qualitative findings using reflective thematic analysis. The reporting guideline for qualitative research was used [i.e., COREQ from Tong et al. (2007)].

### Research team

The focus groups were conducted by the first author, a white, female, hearing, specialist SLT and clinical doctorate fellow with 12 years of experience with working with deaf children and their families, and the second author, a white, deaf, female, QToD and consultant in deaf education with 34 years of experience with working with deaf children and their families. The first author was the lead facilitator and the second supported the facilitation. Both attended training in conducting online focus groups from the Social Research Association in the UK.

Though the first and second authors were perhaps known to the professionals (working in the same field), no close personal relationships were established. This meant that professionals did not assume the authors knew anything of their work or their experiences. The importance of the moderators knowing the topic, the culture and traditions is essential (Litosseliti, 2007), nonetheless professionals were encouraged to be explicit with their reasoning, as though the moderators were new to the field, to avoid the researchers inferring meaning. Professionals were aware of the short-term aims of the research: to explore and explain findings in the e-survey, and the long-term aim: to develop an evidence-based clinical assessment tool.

### Recruitment

The following professionals with any level of experience in working with deaf 0 to 3-year-olds and their families were invited to fill in the e-survey: SLTs, QToDs, Auditory Verbal Therapists (AVTs),^1^ Psychologists/Psychiatrists, and professionals working in Deaf Child and Adolescent Mental Health Services (DCAMHS). In the e-survey information sheet, participants were informed of the follow-up focus groups and invited, using a separate link, to register their interest in participating in the focus groups. Participants shared their contact details and some demographic information (profession, geographical location, hearing status, years of experience, gender, and ethnicity). The separate link ensured that e-survey responses remained anonymous. Forty-two professionals registered their interest in participating in the focus groups.

### Sampling

Sampling was initially intended to be informed by survey findings, however survey analysis found no differences in PCI assessment practices between professionals’ roles, hearing status, languages used at work, or years of experience (Curtin et al., 2023). Therefore, registered professionals were purposively sampled based on the demographic identifiers listed above to ensure diversity, inclusion, and a range of perspectives. Twenty-three professionals were emailed an invitation to the focus group, and the information sheet and consent form.

### Sample size

Nineteen professionals originally agreed to participate, with one drop out. As focus groups were online and involved 14 hearing and four deaf professionals using their preferred languages, i.e., English or British Sign Language, the group size was slightly smaller than usual. Carlsen and Glenton (2011) found the average sample size is eight participants There were two groups of four professionals, and two groups of five.

### Professional demographics

Professionals from each of the four focus groups are shown in Table 1. Most professionals were white, hearing, female, and QTODs. Whilst there was a range of years of experience and geographical location, most professionals had over 20 years’ experience and were working within the south of England. There were two SLTs practicing as AVTs.

**TABLE 1:** Whole group characteristics (*n* = 18).

Sex	Female	94% (17)
	Male	6% (1)
Profession	QTOD	55% (10)
	SLT	39% (7)
	DCAMHS Professional	6% (1)
Hearing Status	Hearing	78% (14)
	Hard of Hearing / Deaf	22% (4)
Years of Experience	0–3	11% (2)
of 0–3 year olds	4–10	28% (5)
	11–15	17% (3)
	16–20	11% (2)
	+20	33% (6)
Geographical Location	England South	38% (7)
	England NE	17% (3)
	England NW	17% (3)
	Scotland	11% (2)
	Wales	11% (2)
	Northern Ireland	6% (1)
Ethnicity		
	East Asian	6% (1)
	White African	6% (1)
	White English/ Welsh/ Scottish/ Irish	82% (15)
	White European	6% (1)

### Setting

Due to the Coronavirus-19 pandemic, all four focus groups were conducted online via Zoom software. Professionals joined the meeting from work/home in a private room. Due to a range of hearing and deaf professionals attending these groups, there were between one and four non-participants in each meeting (technical support, closed captioners, and qualified British Sign Language (BSL)/English interpreters).

### Topic guide development

This project is supported by a patient and public involvement (PPI) group of nine hearing parents of deaf children and eight hearing and deaf professionals, who collaborate with the first author as research partners and experts by experience. For the current study, the quantitative survey results (Curtin et al., 2023) were shared with the PPI group who co-created a topic guide (Supplementary Appendix A). The PPI group wanted to understand the motivations for assessing PCI, gain clarity on how professionals perceive the importance of the top ten skills identified in the survey (Supplementary Appendix B), and see if relationships existed across the parent behaviors. The PPI group raised the lack of an evidence base for families who use a home language other than English and families with children with additional needs. Questions were therefore created to probe best practices in relation to these two populations. Half of the professionals in Curtin et al. (2023) reported they regularly asked parents about their wellbeing. However, the PPI group experiences suggested this was not common and therefore it was pursued in the focus groups. Lastly, Curtin et al. (2023) reported that a quarter of professionals did not always set goals after PCI assessments, so the PPI group wanted to explore goal setting in more depth. Parents in the PPI group were particularly keen to hear what professionals do with assessment data as not all parents had experienced receiving feedback or goals following a PCI assessment.

### Data collection

Professionals were sent the topic guide a week before attending their focus group. Each focus group lasted 90 min. All focus groups were recorded and then transcribed. Field notes were made during the sessions by first and second authors, to clarify understanding and note non-verbal expressions (e.g., head nodding, clapping).

### Ethical considerations

Ethical approval was granted from City, University of London’s School of Health and Psychological Sciences Research Ethics Committee (ETH2021-0335). Professionals were not asked high-risk or controversial questions and questions remained focused on PCI assessment practice. Professionals gave their consent for direct quotes to be used in publications with real names redacted to protect confidentiality. All attendees (including non-participants) committed to a promise of confidentiality. To acknowledge their commitment, professionals were sent a £25 ‘thank you’ voucher.

### Data analysis

All responses (spoken English and interpreted BSL) were transcribed into written English by either a live closed captioner or automatic transcription (i.e., OtterAI). The first author listened to each recording and made corrections to ensure accuracy. For initial coding, software NVivo 12 was used by the first author.

A seven-phase approach to reflexive thematic analysis was used to analyze the data (Braun and Clarke, 2022). Transcribed data were listened to during accuracy corrections, read and re-read, and then features of the data set coded. The first author clustered codes together into initial themes and presented these themes in a series of ‘case by code’ matrices, i.e., each participant was a case, not each focus group. These matrices (along with the transcripts) were then shared with the authorship team for review and refinement. Many of the smaller themes were pooled to create richer exploration of overarching topics. Themes were defined and named, and illustrative quotes were decided upon before producing a final report and coding tree (see Supplementary Appendix B). Participants names were replaced by labels linked to their profession and hearing status, e.g., “hearing SLT 1,” “deaf QToD 1.” Codes and themes were independently verified to ensure reliability of results between the first, second, and last author.

### Reflexivity

We remind the reader this study aimed to provide further explanation to our quantitative survey results. The authorship team’s thematic analysis therefore had an inductive, semantic, and experiential orientation to the data. This means that coding and themes were organically driven by data, stayed close to the participants’ language, and had an essentialist approach, i.e., the analysis aimed to capture truth and reality from within the participants’ contributions - ‘a hermeneutics of empathy’ (Braun and Clarke, 2022, p.160). That said, the authors had a critical realism ontology that postulates a reality that exists beyond the researcher’s ideas, but also recognizes that the researcher is part of the world they are aiming to analyze, and that ‘human practices always shape how we experience and know’ (Braun and Clarke, 2022, p.168).

The first author and main contributor to data analysis acknowledges her own ‘situatedness’ as an insider researcher, a member of the group being studied. She is hearing, uses spoken English as her preferred language and is proficient in using British Sign Language (certified to level 6). She has much experience of working collaboratively with deaf QToDs and sign language instructors. She has also attended a foundation course in listening and spoken language (at Auditory Verbal UK). These experiences and courses mean that she values spoken and signed languages and for many children aged 0–3, recommends a bilingual / bimodal communicative approach for a multitude of cognitive, socio-emotional and language reasons. This perspective will have influenced interpretation of the findings below. In addition, the first author will have also been influenced by the aforementioned PPI group when the findings were shared with them. There was strong support for family-centered (not child-centered or mother-centered) assessment, with opportunities for sensitively given, direct feedback on PCI, provided at a pace that considered family readiness. Some of the PPI members’ experiences were different to the findings in the paper; they reported both a lack of PCI assessment feedback and information sharing between professionals.

## Findings

**FIGURE 1:**
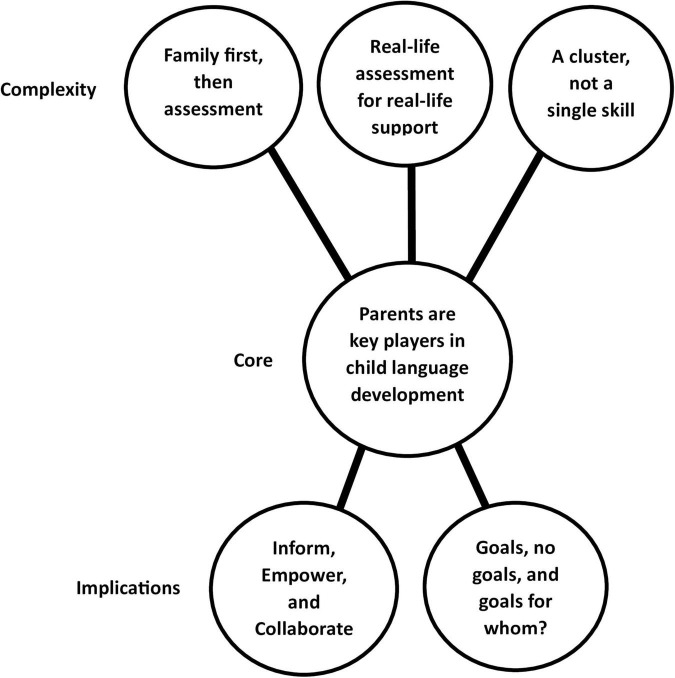
Key themes illustrated.

Six themes were generated from the data and illustrated in Figure 1. A coding tree (Supplementary Appendix C) presents the journey toward each theme in relation to the research questions. Unless specifically stated, all professions shared similar views; due to manuscript length, this cannot be illustrated using dual or multiple quotes, therefore single profession quotes have been featured.

### Parents are key players in child language development

This theme speaks to how central parents are to a deaf child’s language development and why parents are important to assess. As their child’s most frequent and influential interlocutors, parents have the greatest impact on their child’s developmental outcomes. Parental use of helpful communicative behaviors leads to successful language learning. Observing a positive attachment between caregiver and child and how this can support positive social and emotional development was also regularly discussed.

‘So much research has shown the importance of parental involvement and how that leads to better outcomes for deaf children… That’s why we observe parents in the home 0–3, because that’s an important age and when they get most from the language input’ **– deaf QTOD 2**

‘When those important people use good interaction strategies around children then the outcomes are generally better’ **– hearing SLT 1**

For many parents, interacting with a deaf child is a new experience and requires a shift in how they might typically engage with their child. Professionals therefore deemed it is necessary to assess (or observe) parents’ skills to get a full picture of interaction at home, to ‘know what you’re working with’ (hearing, AVT 1), to ensure that language learning is at optimal, and to know where or how to provide support.

‘For me it’s connected with language. A lot of why I am observing is to see how this parent is interacting: Are they using visual strategies? Are they getting their child’s attention?… It’s important that they are learning to interact in that new way, in a visual way with their child, so [the child] can learn that language and have access to language’ **–** **hearing QTOD 1**

Efficiency in the use of professionals’ limited time and resources was a key driver in why assessing parents’ behaviors was considered important. Professionals described educating and upskilling parents as an ‘investment’ as they were potentially preventing further professional support later in the child’s life.

‘It doesn’t matter if we are the most amazing professionals in the world, and even if we have the luxury of having quite high input. If we see a child once or twice a week for an hour, it’s a drop in the ocean in that child’s life… If we can promote and build on strategies for good parent child interaction, that is what is going to make the difference’ **– hearing QTOD 2**

Assessing PCI had the added benefit of showcasing progress, both with the parent, but also with colleagues and line managers, where demonstrating accountability to had importance.

‘I think it’s also to qualify your time and the interventions, certainly in our area, they love a bit of data that shows progress and evidence of development’ **- hearing TOD 3**

Two QToDs shared historical resistance from managers for centralizing parents in their work rather than working directly with the child during the early years. One said ‘as far as they are concerned, we are teachers and we work with children’ (hearing, QTOD 3). They had spent considerable amounts of their time highlighting the evidence and financial gains of working with parents to legitimize working in a family-centered way. By contrast, SLTs did not report having that same barrier; PCI was quite a common feature to assess for many communication conditions in the early years.

### Family first, then assessment

This theme represents four important factors that must be known, discussed, and established before embarking on a PCI assessment with a parent. These prerequisites were: culture and language of the home; general knowledge of child development and specific knowledge of each deaf child; parental wellbeing; and the parent-professional partnership.

#### Culture and language

Knowing which languages were used between the family, and which languages (if different) were used with the deaf child was important information. Heterogeneity exists across families as interaction behaviors will be influenced by their culture and context.

‘Eye contact and looking at people’s faces is culturally dependent… I think it’s important to acknowledge that you might need to change things because the child is deaf… but you need not assume something is wrong because you’re not seeing it. Actually, the child could be behaving completely appropriately within the bounds of what they see in their family’ - **hearing, SLT 3**

Working with interpreters and bilingual support workers was discussed, with a preference for the latter. Benefits included smooth communication between the professional and the family and increased skill in working effectively with a range of families due to the professionals’ improved cultural competence.

‘You don’t know what everybody’s culture is like, you can’t have that basis of knowledge for every single culture. So I’m very much trying to work with my colleagues, bilingual support workers. Interpreters sometimes even can give you an idea but you might not base your clinical judgments on it’ **- hearing SLT 3**

It was important that families used their home languages with their deaf child, so that parents felt comfortable, but also so that the deaf child was exposed to a rich, grammatically correct first language. Professionals acknowledged parental anxiety about doing this due to fears their deaf child would be unable to learn two spoken languages.

‘We do encourage parents who have English as an additional language to use the home language because they [the child] will be able to learn other languages more easily… I have parents saying to us “we don’t want to teach them our home language, we want them to learn English because that’s what they’ll be learning at school,” and it’s trying to kind of turn that on its head and say “well actually, it is important to learn your home language as well as English, if they learn the home language first, English will happen.” But sometimes it’s convincing the parents of that.’ **- hearing QTOD 7**

A diverse case was encountered within these discussions where one professional shared great concern with the blanket approach that all deaf children from non-English speaking families should learn the spoken language of the home. This professional felt that success in one or two spoken languages was dependent on hearing level and that severe to profoundly deaf children could struggle with this. They stressed the importance of access to a visual language for deaf children, especially for severe-profoundly deaf hearing aid users, or for deaf children who do not use any audiological devices, and raised the risk of language delay if the above was not considered.

When assessing PCI with families who use spoken languages other than English in the home, professionals encouraged parents to use songs, books, games, and other customs from their languages and culture. During these PCI observations, professionals described ‘stepping back’ and observing more; they looked at how the parent was using the home language and how they were engaging with their deaf child. Observations could still be made of the parents’ interaction skills and whether the child was responding with babble or imitations of the parent’s facial expression, gesture, tone, rhythm, or speech sounds.

#### Knowing the child

Knowing the ages and stages of child development and early communication enabled professionals to be more focused in their observations, e.g., noticing milestones as well as missing skills, or knowing the next step in development the child may be working toward. Learning about *each* child referred was discussed, i.e., their medical needs, emotional needs, additional diagnoses, level of deafness, use of technology, and whether any other professionals were involved.

‘For the child to achieve their linguistic and educational milestones…we need to look at the whole picture, their wellbeing, not just solely at their deafness and seeing them as someone who can’t hear. We need to think of them holistically’ **–** **deaf DCAMHS professional 1**

For deaf-plus children, knowing about their use of technology allowed for a better understanding of the child’s access to, or perception of, language and interaction.

‘For those children [with complex needs] you need to get as clear audiological information as you can… audiology and fitting of hearing aids is often left later than it should be, or it’s not pushed quite as well… For many of those children, that might be the sense that they are dependent upon’ **- hearing QTOD 4**

One professional shared her experiences of working with a deaf, partially-sighted child and how dual sensory needs impacted on both attention getting and on maintaining joint engagement. Deeper knowledge of the child allowed for a more context-specific, family-focused assessment of the deaf child and their family.

PCI assessments including deaf-plus children tended to be more child-focused where the child’s stage of development was sensitively considered using developmental trackers such as ‘Success from the Start’ (National Deaf Children’s Society, 2020). Professionals observed the methods used by the child to signal communicative intent, as these might differ from a typical trajectory. Parent interaction behaviors assessed would be the same, but the rate and pace at which new skills were expected or encouraged would be set by the child.

‘It’s looking at the child rather than the parents’ interaction… and I think that can come down to very, very small, fleeting moments… Being able to assess the interaction and pick up on those points, and then highlight those for parents to build on… It is more complex, it’s about breaking those stages down into very, very small parts depending on the child’s needs… I think it is about working through the same sort of stages, the same skills, but just at a different pace… at the child’s pace’ **- hearing SLT 5**

#### Parental wellbeing

If concerns about acceptance, early bonding, or self-efficacy existed, professionals would prioritize support around these areas. A confirmation of childhood deafness can be difficult for parents and spark additional emotions such as guilt, of not being good enough, and of not feeling skilled in how to communicate. For some, how they were told about their child’s deafness influenced acceptance, as it was negatively framed from the beginning.

‘The very most important thing to talk about is the parents’ feelings about their child… rather than the hearing aids or how they work… to make sure they’ve got that initial bonding and they’re enjoying their experience with their baby…. I do experience a few tears within the first visits and I’m okay with that. I think it’s a relief you know, parents sometimes need to express their emotions and bring out how they feel before they can move on’ - **hearing QTOD 6**

Consideration of parent readiness, and of the parent’s emotions should always be in the foreground and set the pace for any assessment and/or intervention plans. Providing unbiased information, that may need to be repeated, was important.

‘Some parents want to understand what does the audiogram say? Do the hearing aids make a difference? Will they always be deaf? Or was it my fault? Or, you can also have where they don’t want to know anything about the genetics or whatever, and they’ll say ‘We are happy to accept our child. We love him as he is’… You really have to read it, go at the family’s pace… be in tune with them’ - **hearing QTOD 4**

‘I think we try and make sure that our families are happy to ask us the same questions again and again… So you’re the gateway into this world, but I think we really have to be very careful not to be the gatekeeper into the world, we just need to make sure everybody has all the information’ - **hearing SLT 5**

Ways to check-in on, and support parental wellbeing included informally observing the bond between the parent and child, asking direct open questions such as “How are you feeling about your child’s audiology results?,” being willing to stop and listen to a parent’s struggles if they opened up or shared an experience, and being available to answer questions in-depth. Professionals acknowledged the need for boundaries and stressed the importance of supporting parental wellbeing within the capabilities of their role and skillset. Others felt a need to enhance their own skill development in having supportive conversations, and many mentioned the lack of counseling support services for parents of deaf children.

‘It’s not easy at all I don’t think… because some parents keep it altogether, altogether on the outside but actually when you have left, they are not dealing with it… their mental health is suffering. It’s always really difficult…’ - **hearing, QTOD 5.**

Deaf professionals did not share many experiences of *explicitly* asking parents how they felt about their child’s deafness, but instead three out of four noted that parents seemed to open up and ask them questions about their lived experiences as a deaf child and adult.

‘When I go out to their homes, I feel like, in their environment, the parents are quite relaxed… I do find they like to off load. I don’t know if it’s because I am deaf myself, they feel they can confide in me. I talk about my own experiences, and they really like that’**– deaf QTOD 2**

Professionals recognized that within teams, parents may confide in some professionals and not others and so the sharing of information across teams was important and helpful. This was particularly noted by some SLTs in the group, who felt that QTODs may have better connections with families as they have known the family longer and have more contact time with them.

Providing parent-to-parent support was reported to be another helpful way of supporting parental wellbeing. This ranged from establishing formal parent groups to connecting parents with one another that shared the same culture, language, or case history. Not all parents were ‘group people’ and so professionals’ sensitivity toward parents’ personalities and preferences was required, i.e., a parent who may not be ready for a parent-to-parent meet immediately should be offered another invite later in the support journey.

‘I think sharing experiences with each other is very helpful. I think parents like speaking to other parents rather than the professionals … speaking to another parent is much more relaxing. And you know, there’s always other parents that have been through the same thing and I think they quite like that’ - **hearing QTOD 7**

#### Parent/professional relationship

Establishing a partnership based in support and trust and shared responsibility was important for two identified purposes. Firstly, by getting to know a parent and building a genuine, open and honest relationship, parents began to develop trust in professionals and this could lead to them sharing their worries and concerns. Secondly, a parent who felt safe, supported, and seen as an equal partner was more likely to receive the assessment and ongoing support well and build their own efficacy.

‘Through talking to them, getting to know them and building their trust… the more you get to know a parent, the more you notice if they seem a little off, like they are struggling or stressed… But I think you have got to build that relationship with them so they are comfortable answering those [wellbeing focused] questions as well’- **hearing QTOD 1**

### Real-life assessments for real-life support

This theme describes professionals’ reflections on working with families effectively, suggesting that a ‘real-life’ assessment of PCI enabled ‘real-life’ support that made sense for each family. Observing others, being in the home, observing daily routines, and using video were helpful in accurately capturing the child’s real communication experiences. Joint-working was also discussed in this theme.

#### Not just the mother and deaf child

Many professionals described very busy family homes where there were multiple children present with the parent. Professionals acknowledged the value of observing interaction between only the parent and the deaf child, but also highlighted how unlike real family life this was, as the parent would rarely have one child with them at one time. Instead, real-life dynamics and interactions should be observed at home, so that advice or support following the assessment was relevant, family-centered, and applicable to the family’s situation. The value of observing fathers, other partners, and grandparents was also shared especially if they are one of the deaf child’s regular communication partners.

‘I am working with a family with two profoundly deaf twin babies… and a [hearing] sibling that is a little bit older… From my perspective, it would be ‘how are the interactions’? Because that is what happens all day every day.’ **– hearing QTOD 2**

#### Home is most natural

Observing interaction in the home was incredibly valuable and yielded ‘a gift of information’ (*hearing QTOD 3*). Home provided the most ‘normal circumstances’ for the family to be observed in, representative of everyday life.

‘The home environment is better; it’s a very good environment to observe parents because that’s where they are most of the time. And… for the children, it’s their natural environment’ **– deaf QTOD 2**

Whilst home was the most popular setting for a PCI assessment, many observations also happened at nurseries, clinics, and toddler groups. Each setting provided challenges and benefits with regards to the environment and therefore further insights into the family’s PCI. Some QToDs queried the differences in assessment results generated by SLTs versus QToDs, as QToDs observed parental interaction primarily in homes and SLTs observed interactions primarily in clinical settings. Although, some SLTs had the flexibility to observe the deaf child in a range of settings.

#### Looking at play, and daily routines too

Professionals valued the ‘to and fro’ opportunities between a parent and child that play provided, the range of parental skill that could be observed, as well as the fun, joy, and connection experienced by parent and child. Some professionals warned about arriving and then leaving with a box of toys for the assessment because appraising parents’ interaction behaviors with unfamiliar items demanded even more improvisation and creativity.

Daily care routines such as nappy changing, dressing, mealtimes, bath times, were acknowledged as equally useful to observe. These activities happened with such frequency, particularly with infants aged 0–3, and so provided good opportunities for optimizing parents’ everyday communication skills. When deciding which part of home life to assess, professionals take the parent’s lead.

‘Sometimes you have to be guided by the parents, because… one of the most important things is that parents feel comfortable and that they have their own sense of ability to do this’ **– hearing SLT 5**

For a small number of families with deaf-plus infants, play could be seen as a luxury, particularly if there are multiple medical-based routines to get through in a day. As above, observations with these families might be context bound and family-led, to reduce any burden associated with setting up the assessment.

‘A lot of the parent child interaction occurs around fulfilling those medical needs… I have got lots of these children… A wonderful parent said to me ‘once I make sure I have kept him alive, there is not much time left for play’… For those parents, if you don’t assess it [interaction] within that context, then you could put a lot of extra pressure on them.’ – **hearing SLT 3**

Assessing parent-child interaction was informal. An unstructured, often incidental, observational approach was used either in the home or at parent groups, where the parent might not be aware they were being observed.

‘Obviously we’re working and we’re professional, but at the same time, I’m keeping it in that sort of manner that feels informal and relaxed. I almost observe people without them realizing that are being observed because you naturally just see stuff and think ‘that was brilliant” – **deaf QTOD 1**

#### Video as a window to real life

Using video to capture a PCI assessment had many benefits: greater accuracy; easier to reflect with parents on the skills observed in play-back; opportunities to watch segments repeatedly; the ability to spot things they missed live; the possibility of leaving the parent and child alone so the interaction could be more natural; video provided a measure or baseline for progress; and lastly with permission, professionals could share clips with other members of the child’s Multi-Disciplinary Team (MDT).

‘The benefit was to be able to look back on it and pick it apart. I think it is invaluable, it’s like having that second person there isn’t it? When you can sit back and re watch it, you can look from a removed point of view a bit more’ **- hearing QTOD 3**

Some professionals routinely filmed every session or visit, reporting that parents got used to being filmed and were not aware of the camera after a few sessions. Some services allowed parents to upload or send in their own videos for professionals to view. This flexible provision worked better for some families and children; these videos were often in alternative settings – at the park or at the shops, and at alternative times, e.g., mealtime. These videos offered a more effective or efficient way of capturing multiple aspects of family life and provided professionals with more opportunities to give parents advice and praise.

‘I’ve had some really lovely clips sent to me, interaction clips that have been with grandparents, or with dad to visit his sister… clips without any tension. They’re just what they would do themselves and keep on their phone or show to a friend’ **– hearing QToD 4**

With focus groups held online because of Coronavirus-19, many professionals mentioned the pandemic and the benefits that video use/telehealth brought. For example, other MDT professionals (i.e., from NDCAMHS or cochlear implant centers) joined the call as ‘silent observers’ and then participated in discussions nearer the end of the session. Those who held a more dispersed national or regional caseload, found being able to remotely ‘enter’ a range of family homes in 1 day incredibly enlightening, providing ‘closer to real life’ (hearing AVT 2) observations than seen at their center or hospital setting. Lastly, remote sessions provided a reality check in terms of each individual family’s set up. During the pandemic, parents were mostly seen at home with all other family members present, therefore a better depiction of weekend or evening life was presented. These ‘real deal’ observations led to a more real or aligned offering of support.

‘It’s been different over lockdown and COVID… I see one child with ANSD [Auditory Neuropathy Spectrum Disorder] that is in a home of 10 children. Seven live at home and it’s only by looking online when we do the Zoom, that you realize how busy, particularly if they say “yes, I’ll see you at six pm,” and there’s literally, people floating you know, pass the whole [uses sign to show people walking passed the camera, uses sign to show busyness] which you don’t get when you go on a home visit because you tend to go to at 11 a.m. or 9 a.m. or whatever, and everybody’s gone to school and it’s definitely given me a totally different insight into what you can also realistically expect when you give advice, because sometimes I think we can be a little unrealistic’ **– hearing QToD 4**

Whilst the consensus was that video was helpful, concerns around the acceptability, practicalities, and implications of video or remote working were raised. For example, some services do not have filming equipment or do not allow its use. Others felt there were legal or safeguarding issues with both the secure sending and receiving of child-based videos, as well as how and where to safely store the videos (both in terms of security and in the practical sense of storage/capacity of servers). Others noted the hindrances of travelling with cameras, tripods, and chargers, but also how arriving with, and setting up, this equipment roused the interest of other children and pets whilst family members were trying to focus. Others remarked that, during the pandemic, seeing family life through a static lens was limiting and they sometimes missed important moments off camera. Further, the pandemic created a barrier to accessing low-income families where the digital divide was most prevalent. Professionals also acknowledged the self-consciousness of some parents and how setting up a camera could be problematic as the activity seemed more formal and parents felt under pressure.

Professionals felt that including video use as part of the proposed assessment’s protocol might help drive change for more resistant local authorities or trusts to allow more filming and encourage discussions about the transfer and storage of videos. Another recommendation to help alleviate these difficulties was to use the family’s recording devices in the home.

‘If they are up for it, use the parent’s phone… I know that means, you then you can’t go away and look at it and think about it, but it’s certainly a way to look at it together. It means you’re very much thinking on your feet but then you bypass the thing about storing video’ – **hearing QTOD 2**

#### Optimal PCI assessment requires joint working

Professionals spoke of effective and less effective joint working in relation to a range of contexts and disciplines. Many QToDs and SLTs discussed working jointly. This included attending child progress meetings, attending home visits to the family together, jointly running a parent group, attending hospital-based appointments with families or hospital-based professionals joining a home-based session. Many professionals acknowledged there was huge overlap in the areas of focus for SLTs and QToDs, i.e., parent wellbeing, audiology, language, and listening, therefore clearly outlining who was doing what was important for joint-working and for families. Key benefits of joint working highlighted by professionals were the opportunity to sound board and ‘bounce ideas off one another’ (deaf QToD 1), deepen clinical discussions, and develop better understanding around the child and family. The benefits of joint working for families included fewer appointments, fewer assessments, less of a burden for the family to repeat information, broader expertise involved with each child’s care, and joined-up, holistic care.

‘Some skills can be very fleeting. I’ve asked other colleagues to look at something and I’ll say, “I think I’ve seen X,” particularly profoundly deaf children with complex needs. And if somebody else can look at it, you know, with a different eye, a speech and language therapist, whoever you happen to work with, that’s really helpful’ **– hearing QToD 4**

Joint working between QTODs and SLTs was less successful when professionals had very stretched caseloads, when there was less deafness expertise, and when teams were geographically spread out. For example, language and communication was a common need for many children living in areas of greater poverty. This impacted QToDs’ and SLTs’ capacity for joint working and joint visits as both were stretched in terms of caseload capacity.

Hearing and deaf professionals regularly discussed the importance and value of working with deaf colleagues. Deaf professionals who were native signers shared a complete fluency of communication with the deaf children they worked with, and self-reported as having deaf identity at the center of their work. They helped to build rapport with the child, to identify the needs of the child and family, and to model successful ways of interacting. It was important that families had access to successful deaf adults both as role models for the deaf child, and in offering the family the possibility to envisage what their deaf child may achieve in the future. Families also learnt and appreciated the value of sign language as an alternative way to communicate.

‘What is important is that the parents have access to both hearing and deaf people. Usually, parents gravitate toward hearing professionals and follow the advice that they get from them, sorry for the terminology, but the idea of ‘curing deafness’… but actually what they need to learn is that language is what is the most important of all. It’s about the child being able to express themselves… Deaf people have the lived experience. They have grown up deaf in this world and so **we** need to talk to them about the journey of language acquisition’ **–** **deaf DCAMHS professional 1**

A lack of deaf professionals who work with deaf children could impact families and professionals’ learning and development, and families’ hopes for their child as they grow up.

‘I think a weakness around the UK and generally, is that at these initial points of contact, these people are not deaf. And personally, I think that that’s wrong. I think it is vitally important to have deaf professionals involved in this whole journey, so that a child and their family can see what kind of person they can grow up to be when they’re older… it’s so important to have role models, to have people being professional, particularly deaf people, because there aren’t enough out there generally working at this level. I think that that should be a basic within services’ - **deaf QToD 3**

### A cluster, not a single skill

This theme describes which parent behaviors professional prioritize in their PCI assessments. Professionals were presented with the top 10 parent behaviors most assessed by professionals during PCI assessments [Supplementary Appendix B: survey data from Curtin et al. (2023)]. Professionals agreed these parent behaviors were beneficial for the development of all languages, signed and spoken, and would therefore be relevant to assess within *any* parent-child dyad, providing the cultural diversity of the family was considered.

‘I think it doesn’t matter. You should have the same approach, the same method and same way of assessing regardless of language’ **– deaf DCAMHS professional 1**

Whilst the behaviors were listed in order of how frequently they were assessed in practice, focus group discussions reflected that these do not necessarily mean they are in order of importance. Two viewpoints surfaced in relation to this. First, that parent behaviors needed to be assessed within a cluster in order to holistically capture the PCI, i.e., observed all at once.

‘What I was thinking was ‘Is there a hierarchy? Are any of those more important than the others?’ When I went down the list, I was trying to think what if I had to put them in order which one would I do first and I really struggled with that, which made me think that most of those on there are of equal importance.’ **- hearing SLT 3**

In contrast, others felt the list was more of a progression: earlier fundamental skills underpinned later, more sophisticated skills. For example, ‘parent is genuinely interested and involved’ (number six in the list) was viewed as a foundation behavior, a potential driver of change and, if not present, the first stumbling block in observing and/or supporting PCI.

‘Without that involvement and interest and emotional availability, I think it’s difficult to focus on any skill’ – **hearing QTOD 5**

The parent waiting for the child to look before communicating (number one) was also raised as the first behavior professionals looked out for because in their view, if the parent was not practicing that, then not much else would be perceived by the child. Many parents may need this skill explained and modeled as they may be unfamiliar to this way of interacting and could easily forget that a child not looking might mean the child was not listening or receiving language.

‘That’s one of the first things that I look at, the parent to wait for the child to look. Besides I just know from my own experience, if someone talks to me without getting my attention, I would miss half of the information. I haven’t got what they said, so I know the importance of eye contact. Parents do sometimes get frustrated because they’re very young to maintain eye contact, children are so distracted, but it’s important to start young and then hopefully it develops’ – **deaf QTOD 2**

Professionals mentioned that the child’s age and/or stage would alter the level of ‘looking’ a parent could or should expect, particularly for younger babies, or infants with additional needs, where head control or neurodiversity could have an impact on successful eye contact. They suggested that ‘face watching is enough sometimes’ (hearing SLT 5).

Another fundamental skill discussed was parental responsivity, a parent following their child’s lead, being attentive to their child’s needs and communicative intents and responding appropriately (number two). Professionals felt if this, often innate behavior was not observed, extra support for the parent would be required.

‘Following the child’s lead is one of the most important ones and goes hand in hand with joint attention… For me they are the core ones that have to be there from the beginning… A lot of the other things kind of follow on from that’ **– hearing QToD 2**

Professionals shared their insights on parents who became good observers of their child, they would notice many or every child contribution to the interaction, particularly early initiations such as eye movements or legs tensing. A parent noticed these behaviors would naturally provide more contingent and effective language input.

Rather than a cluster or progression, a minority group shared an alternative view that the mode of language used by the parents, and the child’s access to spoken language, would dictate where to begin with PCI. For example, if the child was nearly 3 years old, had been bilaterally implanted successfully at 1 year, and was developing age-appropriate spoken English, then face watching or eye contact would not be prioritized, but instead professionals would progress to joint attention on objects whilst listening. However, if a child of the same age was severely deaf, with or without hearing aids, then the parent waiting for the child to look would be the priority and starting point (if not already established).

Professionals identified parent behaviors missing from the top 10 list of most assessed skills. A popular skill raised by many professionals was the parent waiting or pausing to give the child time and space to make an initiation, take a turn, or join in with the play.

‘Does the parent wait for a child to actually start some communication so they have a lead to follow? Rather than thinking it’s the parent starting an interaction all of the time. Waiting is something that helps the parent become more responsive to their child’ - **hearing SLT 2**

Six other skills identified by professionals as missing from the list included: joint attention; the parent engaging in balanced, communicative turn taking with their child; the parent using a range of different word types (nouns, adjectives, verbs) within their interactions; the parent labeling items and offering choices rather than simply giving items; the parent being in an appropriate position to the child; and the parent becoming less intrusive, less directive, and asking fewer questions. Professionals felt these last three skills needed to be explicitly stated as separate skills rather than falling under the category of ‘responsiveness’ or ‘following the child’s lead’ as they currently appeared in the list.

Some professionals felt that educating parents on the concept of language was important, stating that it was important to notice, accept, and encourage any form of first language as this provided a building block to developing future skills in bilingualism, bi-modalism, and or multilingualism. Professionals shared how their services and professional groups were now more focused on the importance of language, communication, and the whole child, rather than sole-focus on listening, spoken language, and speech sound production. There was acknowledgement that this shift in attitude was not universal.

‘All the ToDs and SLTs were pushing for oralism. But then the last 10 years, there’s been a really good shift of attitude. Now we are all in agreement, we’re all focused on language, not just speech, but language, whether it is BSL, or spoken language or bilingualism. We focus on that, and we are all on the same page here’ **– deaf QTOD 2**

### Inform, empower, and collaborate

This theme links to the transformative process that between parent and professionals when reviewing the PCI assessment video. The importance of taking the time to share assessment data with parents was discussed. Parents then understood the purpose of the assessment, became informed, and began to share power and responsibility for their child’s progress.

‘Parents need to be informed. Otherwise, they just feel like they don’t understand, thinking why is this person seeing me?’ – **hearing AVT 2**

‘It gives the parents a chance to see themselves, see for themselves’ **- deaf TOD 3**

Highlighting positive parent behaviors (no matter how prominent initially) and the impact they have on the child’s response or turn was regarded as an empowering activity for parents within the assessment review. It raised parents’ awareness of the behaviors that were supporting their child’s communicative development, and brought suggestion that parents could be the catalyst for change, especially when the importance of the skill was explored in-depth or supported with research.

‘Just reiterating the importance of those [parent behaviors] can be the indicator of change that is needed in order to support the progress… just being conscious that they are doing it is quite key’ **hearing SLT 1**

‘You can pick out some lovely interactions and show evidence to the parent, and show them the difference that it made to their child… Video shows you so much that you don’t see live in the moment’ - **hearing SLT 3**

Gaining feedback from parents was also seen as an important part of the cycle, with many professionals suggesting that parents should lead discussions on their own communicative strengths as well as any improvements they would like to work toward.

‘We are constantly monitoring, feeding back, getting feedback from the parents… that is a really important part of the process. It’s a holistic process, involving the parents…. We would share all we have with parents, and parents are always aware of the purpose of the assessment …we take a very positive approach. We focus on the strengths’ - **deaf DCAMHS professional 1**

Positive feedback empowered the parent to continue using the skills identified and discussed in the assessment review. Parents began to capitalize on their innate skills more consciously. It was felt this strengths-based approach was better than ‘it might be helpful to try this new behavior’ as this was disempowering for the parent and shifted the ‘expertise’ to the professional.

**‘**It [watching back a videoed assessment] shows the parent that they can make progress with their child. You can show the family that they can make the difference. And that’s what we want to do. We don’t want to be showing that **we** can make the difference, we want to show that **they** can make a difference with their child’s progress’ **- hearing QTOD 6**

Professionals noted by regularly offering parents the opportunity to video and review their PCI, parental empowerment developed further. Parents became more at ease with seeing themselves in recordings, and became more skilled and observant in noticing their own behaviors and the impact they had.

‘Just over time, with doing it [videoing interaction] regularly, it becomes much more natural and an ingrained part of early practice. More relaxed conversations of noticing the interactions occur, and it’s a good way of noticing progress’ **- hearing QTOD 6**

Some professionals felt that the review process (informing and empowering the parent) helped to build a trusting relationship between parent and professional. Deciding on next steps would then be done jointly with the parent taking the lead and the professional scaffolding the discussion. This approach ensured the parent embraced the work and increased their engagement and involvement.

‘Being collaborative with parents with your goal setting…. Saying, ‘we’ve seen this, this, and this today… which would you like to do more of?’ Opening it to them. What do you think is achievable? Which one can you most readily apply into your daily routine?’ **– hearing SLT 1**

One professional mentioned that whilst they might have a preferential target in mind for the parent following the review, a collaborative approach was more effective and therefore required ‘being a bit more open minded, sitting back and taking the parent’s lead’ (hearing QToD 6).

### Goals, no goals, and goals for whom?

Explicitly collaborating with parents to decide on parent-focused goals was not a feature of every professional’s practice. This range is explored within this theme. Firstly, most SLTs, both AVTs and the DCAMHS professional described having quite clear parent and child focused aims that were written up, decided upon with parents, shared across the support team, and regularly reviewed. Having goals for the parent would help facilitate change in their young deaf infants.

‘It’s looking at play with purpose. … Where do we need to get to? … Whether you’re a teacher of deaf children or a speech and language therapist, you’re there to help the family progress from one stage to the next… and explain the purpose of what you’re going to be working on next… Now, whether it’s a child with complex needs, and they need to have micro steps, but they need to be there’ - **hearing AVT 2**

Others reported to take a more informal approach, where a goal was considered and noted down by the professional but perhaps not shared with the parent. There was a nuanced belief that perhaps goals were for deaf children, not for their parents.

‘I develop goals, but not, you know, like we would in school… where you have an IEP (individualized educational plan) or a statement or whatever. For me it’s something that I just know. I put in my notes “we are working on this.” It’s not something I am ticking off and assessing in a formal way’ **- hearing QTOD 1**

‘I think a lot of the language type targets are goals and they are written down “this is what we are working towards,” but I can honestly say, I don’t write down ‘these are the targets for the parent in terms of the parent child interaction’ and share that with the parent’ **- hearing SLT 2**

Professionals explained why goal setting for parents can be problematic. One common reason discussed was in relation to families who had deaf-plus infants. These families can often have multiple objectives to work on, and professionals preferred to reduce parental burden and not add to it.

‘I’ve found they often get a lot of targets and goals. Parents may very well decide that they want to focus on one particular area, it might be the physical needs at that particular time… I would actually ask them what they feel their priority is at the moment’ **– hearing QToD 4**

Another reason provided was that goals could be overwhelming for a new parent who has many appointments to go to and many visits to host. Using the term ‘goal’ was debated also, with one professional suggesting less pressured phrases such as “how we can help.”

‘Rather than saying ‘this is going to be the goal’ (because we don’t want to **not** achieve the goal either), we want to make sure that it’s an achievable, fun thing to do with the child, and a natural thing to do in everyday routine… They just can feel overwhelmed, no other parents seem to have goals. You know, I don’t want to make it any different from another parent bringing up a child. I want them to do their communication and language in the most natural way, routine way, throughout the day’ **– hearing QToD 6**

Professionals felt it difficult, forced and unrealistic to set goals with or for families who were not as engaged or open to professionals’ support. Another professional reported difficulty in creating goals with families who struggled to make decisions about their child’s future language or educational setting.

‘They wanted him to be oral, they wanted him to sign. They didn’t kind of stick with anything and it was actually hard to create a goal for them because they didn’t know what they wanted. And every time I tried to suggest something, it was kind of “no we don’t want that,” but they didn’t have an idea of what they did want’ **– hearing QTOD 7**

Some parents liked monitoring their child by using developmental journals such as ‘Success from the Start’ (National Deaf Children’s Society, 2020). After mapping out their child’s current stages of development, professionals would engage in informal discussion around the activities the parent could do with the child, but no parent-focused goals would be set. Conversely, some parents disliked developmental trackers as it highlighted skills not yet achieved by their child. Whilst all professionals seemed to be working toward progressing the deaf child and their parents, goal setting was a ‘case by case thing, depending on where the parent was at, and what would work well for them’ (hearing QToD 1).

## Discussion

This study aimed to explain why and how early interventionists working with deaf infants aged 0–3 assess parents’ interaction skills as part of their practice. Hearing and deaf professionals attended focus groups, steered by a topic guide that was influenced by a large UK survey (Curtin et al., 2023) and co-produced by the authors and a patient and public involvement group.

This study set out to understand the importance of assessing parent behaviors. Professionals placed parents at the core of their rationales for assessment; they were well-versed in the evidence base, they acknowledged how central parents are for language development, how expert they are in understanding their own children, and how parent involvement should be a core focus of professional practice. Parents and primary caregivers are known to be important for language learning (Rowe, 2012); children must be exposed to language to learn it. Recent PCI research in hearing dyads reports that levels of language exposure and conversational turns between parent and child impact language processing over and above quantity of words (Romeo et al., 2018). Houston (2022) argues that the association between language input and language outcomes for deaf and hard of hearing children are more complex because of four differences: total language input; accessible language input; attended-to language input; and language co-ordinated with cognitive level. Houston recommends that early interventionists enhance parents’ knowledge, self-efficacy, and skill so that each deaf child receives accessible, developmentally appropriate language in their family context. As such, it seems paramount that professionals assess parents’ interaction skills to know where in the language input framework to begin providing support.

Informing, empowering, and collaborating with parents was the most important and powerful theme and implication generated from the data, and aligns with the recommendations made in the Family-Centered Early Intervention (FCEI) consensus paper by Moeller et al. (2013). In the present study, professionals said that by educating parents in the assessment process before it begins and then taking time to review the assessment together, an informed, empowered, and conscious parent developed. The parent was more aware of how their interactions could influence their child’s language development. Parental self-efficacy (parents’ beliefs about their ability to successfully perform in their parenting role) has been shown to lead to improved maternal language input (DesJardin, 2006; DesJardin and Eisenberg, 2007) and child language development (Niparko et al., 2010; Cruz et al., 2013). Further, in the current study professionals said that by both parent and professional focusing on the positives seen in assessment, parents experienced specific positive feedback on skills they already have. This aligns with Szarkowski and Brice’s (2016) Positive Psychology Framework, where parents are encouraged to think of the positive, joyous experiences that come with parenting a deaf child. When they did, parents reported a transformative effect, whereby simply spending time in the joy of their child and the parenting process, parents felt positive, grateful, and experienced growth. Davenport et al. (2021) reported that parents need to feel they are competent and capable in their role as language models to fully enhance a deaf child’s language growth. This current study suggests this shift can be gained through assessment, shared review, education, coaching, collaboration, and time with deaf professionals.

Another aim of the study was to understand how PCI assessments influence professionals’ practice. Professionals said that reviewing the PCI assessment together fostered a balance of power and shared responsibility. This shared focus, shared analysis, and shared drive to make progress led to collaborative decision-making for goal setting and intervention planning. Whilst it was clear most professionals practiced this way, there was some divergence between the professionals in the focus groups. Interestingly, this was also seen within the survey data, where 76% of professionals *always* created goals and 24% *sometimes* did (Curtin et al., 2023). Professionals in our study showed sensitivity to not creating goals where parent readiness, acceptance and engagement was not achieved. In the wider, hearing literature on PCI, parents also report on their needs in terms of readiness (i.e., child and family preparedness, acceptance, and capacity to take part), with a view that their engagement is facilitated through a supportive parent-professional relationship (O’Toole et al., 2021). Prior to setting goals and considering intervening, it is recommended that professionals attune to parental wellbeing, and discuss parent’s expectations and involvement (Levickis et al., 2020). Goal setting with parents is a prominent feature across many disciplines, e.g., physiotherapy and occupational therapy (Vroland-Nordstrand et al., 2018; Harniess et al., 2021) and their ‘attainment’ is often linked to motivation and engagement. In the current study, there were some queries around who goals should be for (i.e., children or parents). Goal-focused conversations are generally accepted as tools used for skill improvement and behavior change (Schenk et al., 2023) and are therefore essential in helping parents (the people within PCI with the greatest capacity to change) to adapt their behaviors in interaction.

Across four of the remaining five themes was the acknowledgement of great complexity when observing PCI within real-life, family life contexts. When investigating how a best-practice assessment of PCI might be conducted, professionals argued for ‘true to life’ assessments that acknowledged factors often unmentioned (or excluded) in research such as the influence of siblings, multiple caregivers, the home environment, parental wellbeing, deaf-plus children, and / or families who use languages other than English. Further, professionals advocated for PCI assessments of play *and* daily routines, in order to fully capture what happens in the home environment.

Professionals agreed it was impossible to select the single most important skill to assess in PCI and that it was similarly difficult to assess any one skill in isolation from others. They agreed that the top ten most-assessed parent skills generated from the survey (Curtin et al., 2023) were beneficial for all languages and all needs. Parental engagement and sensitivity were given particular mention across the focus groups, perhaps because of their well evidenced importance in PCI with deaf infants (Vohr et al., 2010; Ambrose, 2016). Face watching and eye contact were also picked out from the top ten list. Most SLTs and QToDs included these visual behaviors in their PCI assessments, as they regarded them important for learning sign and or spoken language. A small group of professionals in our study focused less on these. Professionals felt joint attention and balanced turn taking between parent and child were core behaviors missing from the top ten list. This also aligns with multiple studies that have shown the positive relationship between deaf children’s language scores and time spent in co-ordinated (or mutual) joint engagement (Gale and Schick, 2008; Cejas et al., 2014; Dirks and Rieffe, 2019).

Because professionals were unable to select the most important parent behavior to assess, they used multi-simultaneous skill observation to analyze PCI. This is in stark contrast to the PCI research base on deaf infants, where mostly one or two features of parental interaction are under the microscope, the context is play, and participants are mostly monolingual, mother-child dyads, observed in labs (Curtin et al., 2021). Whilst this is the majority, there are notable papers that include father-child dyads (Loots and Devisé, 2003; Loots et al., 2005; Wille et al., 2019) and report on a range of language used between parent and child (Vohr et al., 2010). These findings suggest a need for ethnographic research conducted in the home environment, observing *families* (not just mothers and their deaf children) during a range of daily activities, throughout a day, or week, or perhaps longer. Research undertaken with hearing families [see the systematic review by Holme et al. (2022)] increasingly features researchers capturing interactions within daily routines, in homes, using video and audio-based recording equipment. There are some recent studies with deaf infants that seek to capture interactions throughout the whole day (Brock and Bass-Ringdahl, 2021) or during activities such as mealtimes (Smolen et al., 2021) but these embrace audio-only recording software (LENA belts) which therefore limits the perspective on PCI with deaf infants.

For deaf-plus children, another layer of complexity was reflected. Professionals reported a slightly adapted approach where the professional needed to know the child’s additional condition(s) and how their development, in association with their deafness, might be impacted. Professionals felt that these families needed to have a more child-focused assessment where parent and professional looked at the child’s communicative intent first, rather than the parent’s behavior. This shift in assessment focus aligns with research from Turner Dougherty and Day (2022), who also suggest that families with deaf-plus children require an independent lens for assessment and intervention. Professionals in the current study also felt that assessment and support needed to follow the same trajectory as typically developing deaf children, maintaining high expectations, but at a slower pace. Lastly, the goal-setting stage needed strong collaboration with parents as a communication focus may be lower on their priority list, when compared to nutrition or physical needs. Parents of deaf-plus infants can feel less confident in their parenting skills due to the complexity of their child’s needs and can also feel as though they have less involvement in their child’s daily activities, perhaps due to the high number of services planning and participating in their child’s schedule (Turner Dougherty and Day, 2022). The ‘inform, empower and collaborate’ findings are even more essential with this group, ensuring that parents feel confident, involved, and in control of making informed decisions about their communication behaviors and goals. ‘Complex Needs, Complex Challenges’ (McCracken and Pettitt, 2011) reported the lived experience of 50 parents of deaf-plus infants and made a series of recommendations for professionals on assessment: provide rigorous and timely assessments; ensure parents are made aware of the purpose and findings; actively include parents in the assessment process as they can help build a picture of needs; value the importance of parental expertise; offer a flexible approach in terms of re-assessment and location; provide effective, coordinated care between all multi-agency teams – sharing information between services as well as parents; and discussions about approach should follow assessment. Many of these recommendations aligned with our professionals’ contributions on best practice with this population (and with deaf children more generally).

A final layer of complexity mentioned by professionals was supporting families with deaf infants who do not use English at home. The need for culturally competent and responsive services is necessary in deafness, where respect to each family’s cultural and linguistic diversity is given (Yoshinaga-Itano, 2014). Successful ways of working within multilingualism included finding out about the languages used and the culture of the home, involving bilingual co-workers and interpreters in sessions, encouraging the home language be used with the child, and encouraging songs, books, and games from the family’s culture to be used during PCI assessments in the home. During PCI assessment, some professionals reported a ‘stepped-back’ approach. This meant professionals were looking more to the visual parent behaviors within engagement and parental sensitivity (face watching, joint attention, warmth, genuine interest) and then relied on colleagues for language content and cultural brokering. Research into the language outcomes of deaf multilinguals is scarce. A review of 22 studies on this population (Crowe, 2018) produced diverse results due to the range of ages, languages and domains of speech and language development assessed. Studies found deaf multilingual learners had better outcomes (in speech perception, Sininger et al., 2010), similar outcomes (in speech production, Bunta et al., 2016) or worse outcomes (in vocabulary skills, Deriaz et al., 2014) than their comparison groups. With such a limited evidence base and variable outcomes, it is not surprising that, within our study, some tension existed in the advice that professionals give parents, but mostly professionals felt it was important to promote multilingualism.

A way of successfully negotiating these layers of complexity was multi-professional joint working. Professionals were able to list many benefits of joint-working such as being able to sound-board off one another, being able to build a more holistic view of the family and child, building a better suited package of care for families, and reducing the burden on families to repeat assessments and/or repeat information. Holzinger et al. (2022) recommend regular multi-professional assessment and monitoring as a way of ensuring that each early interventionist is offering support that is effective in strengthening the functioning of the family, supporting wellbeing, and building capacity in parent-child interaction strategies. In our data set, many hearing and deaf professionals were able to identify the merits of working with and learning from deaf QToDs, Deaf Language Specialists (Hoskin et al., 2023), and/or deaf CAMHS professionals. Deaf professionals have lived experience, a unique way of perceiving, making sense of, and supporting complexity too. Known benefits for involving a range of deaf adults within family-centered early intervention include reductions in parental stress and increased confidence (Hintermair, 2000), the opportunity for parents to envisage success for their children (Rogers and Young, 2011), parents learning a range of visual strategies to assist with language learning (Humphries et al., 2012), and deaf adults being role models for families and deaf children (Cawthon et al., 2016; Gale, 2021). Our data suggests another benefit; parents seem to have a willingness to open up, ‘offload’ and confide in deaf professionals, without prompt questions around their wellbeing.

A resource regularly mentioned by professionals to aid with accurately capturing busy, multi-layered, family-child interactions was the use of video. Video, as many professionals shared, offered a chance to capture complexity and shine a light on real life. It also provided the opportunity to freeze-frame multifaceted moments and reflect upon them with parents, accounting for multiple behaviors simultaneously. Playback of short video segments or ‘thin slices’ of interaction to represent a parent and child’s natural pattern of interaction is a well-used, well-evidenced methodology, especially within Video Interaction Guidance Therapy (Landor et al., 2011). Video recordings provide an opportunity for ‘micro-analysis’ (Trevarthen, 1980) and partnership working with parents (Cummins, 2021). Its use for outcome measurement in parent-implemented interventions is well documented in research with hearing (O’Hara et al., 2019) and deaf populations (James et al., 2012; Lam-Cassettari et al., 2015; Ambrose et al., 2023). Video feedback is often recommended as a tool to use in family-centred early intervention with deaf infants (Mood et al., 2020) and from our data set, it would seem that this is because video allowed a parent to ‘see’ and seeing led to understanding which then led to empowerment and behavior change. Whilst most of our study’s professionals were aware of the benefits of video use, it was clear that not all services use it. Each of the APEASE criteria has relevance here (Michie et al., 2014): Affordability (not all services could afford the equipment and/or resources to record and store video data); Practicability (transporting and setting up the devices brought challenges, and static cameras during telehealth appointments limited access); Effectiveness (queries were raised around representativeness, i.e., parental behavior changed once being videoed); Acceptability (parents, professionals and/or managers avoided or rejected video use, deeming it not useful or important); Side-Effects and Safety (issues around the sharing and storage of videos of young children); and Equity (a mention of the digital divide during the Coronavirus-19 pandemic).

## Limitations

The professionals recruited were self-selected volunteers, and likely passionate about PCI and early years work, therefore a level of bias may be present in our findings. Secondly, focus group data are reported practice, not ethnographic, observational research (which would provide purer insights into practice), so what professionals say they do, may not completely represent actual practice. Thirdly, an attempt was made by the authors to purposively sample professionals on protected characteristics, there was, however, an underrepresentation of male professionals and those from ethnic minority groups.

## Implications

For professionals working with families of deaf children and for educators providing their training, our findings suggest four points to consider when assessing PCI. Firstly, be family-centred in approach, ensure that time is taken to get to know the family context, the child, and check in on parental wellbeing at the beginning of care. Embrace the busyness of family life, of cultural diversity, of daily routines, and of siblings. Secondly, invest time in developing a positive, supportive, non-judgmental relationship with parents. Thirdly, inform and empower parents through the use of video, consider its merits for capturing all the finite details, and providing a source of reflection for parents on playback. Lastly, PCI assessment should lead to jointly discussed and agreed plans for progress. For researchers in the field of deafness, there is a clear need to observe and analyze complexity: encompass activities of daily living beyond the luxuries of play and book reading, include multilingual deaf learners, include deaf-plus infants, observe multiple parent behaviors in homes, and use and analyze video recordings.

## Conclusion

This qualitative study provides insight into the mechanisms and motivations for professionals assessing the interactive behaviors of parents who have deaf children aged 0–3. Professionals considered the parents’ role as core for deaf children’s language acquisition and a worthy investment of time. Professionals used assessment to understand where to start with a family and to show progress. Before undergoing PCI assessments, professionals recommended providing holistic care where time was taken to understand the family context and support parental wellbeing. Reviewing video-recorded PCI assessments with parents was highlighted as a transformative way to inform, empower, and collaborate with them. Professionals acknowledged that family life is multi-faceted, but support is most meaningful to families when professionals worked with these differences and incorporated them into assessment, goal setting, and intervention plans.

## Data availability statement

The raw data supporting the conclusions of this article will be made available by the authors, without undue reservation.

## Ethics statement

The studies involving humans were approved by the City, University of London’s Language and Communication Science Proportionate Review Committee (ETH2021-0335). The studies were conducted in accordance with the local legislation and institutional requirements. The participants provided their written informed consent to participate in this study.

## Author contributions

MCu: Conceptualization, Data curation, Formal analysis, Funding acquisition, Investigation, Methodology, Project administration, Resources, Visualization, Writing—original draft, Writing—review and editing. TW: Data curation, Investigation, Validation, Writing—review and editing. RH: Conceptualization, Methodology, Supervision, Validation, Writing—review and editing. GM: Supervision, Writing—review and editing. MCr: Conceptualization, Formal analysis, Investigation, Methodology, Supervision, Validation, Writing—review and editing, Project administration.

## Footnotes

1 In the UK, AVTs were Teachers of the Deaf, Speech and Language Therapists and/or Audiologists before they qualified as Listening and Spoken Language Specialist Certified Auditory Verbal Therapists.
