# Supplementary material for: “It doesn’t matter if we’re the most amazing professionals in the world…” A qualitative study of professionals’ perspectives on parent-child interaction assessment with deaf infants
Source: Front Psychol. 2024 Mar 4;15:1315220. doi: 10.3389/fpsyg.2024.1315220 (PMC10944883; doi:10.3389/fpsyg.2024.1315220)
Supplement: Supplementary file 1 [file Table_1.DOCX]

***Appendix B: Top Ten Parent Behaviours Assessed in PCI (n=155) from Curtin et al., 2023***

| **Parent Behaviour Assessed** | **%** | **(n)** |
| --- | --- | --- |
| Parent waits for the child to look | 82.6% | 128 |
| Parent is contingent and responsive, follows their child’s lead and responds with on-topic behaviours or language | 69.0% | 107 |
| Parent uses multi-modal strategies to gain the child’s attention | 59.4% | 92 |
| Parent ensures they are face to face with their child | 58.1% | 90 |
| Parent is stimulating and can provide appropriate pace, play, and language structures | 54.2% | 84 |
| Parent is available to the child, genuinely interested and involved | 53.5% | 83 |
| Parent expands their child’s language by adding 1 or 2 new words/signs | 50.3% | 78 |
| Parent uses child-directed language (spoken or signed) to raise child's interest | 49.7% | 77 |
| Parent interprets their child’s behaviour with language (spoken or signed) | 45.2% | 70 |
| Parent comments on, or describes the child’s action | 41.9% | 65 |
