# Supplementary figures and images for: “It doesn’t matter if we’re the most amazing professionals in the world…” A qualitative study of professionals’ perspectives on parent-child interaction assessment with deaf infants
Source: Front Psychol. 2024 Mar 4;15:1315220. doi: 10.3389/fpsyg.2024.1315220 (PMC10944883; doi:10.3389/fpsyg.2024.1315220)

***Appendix C – Coding Tree***

***
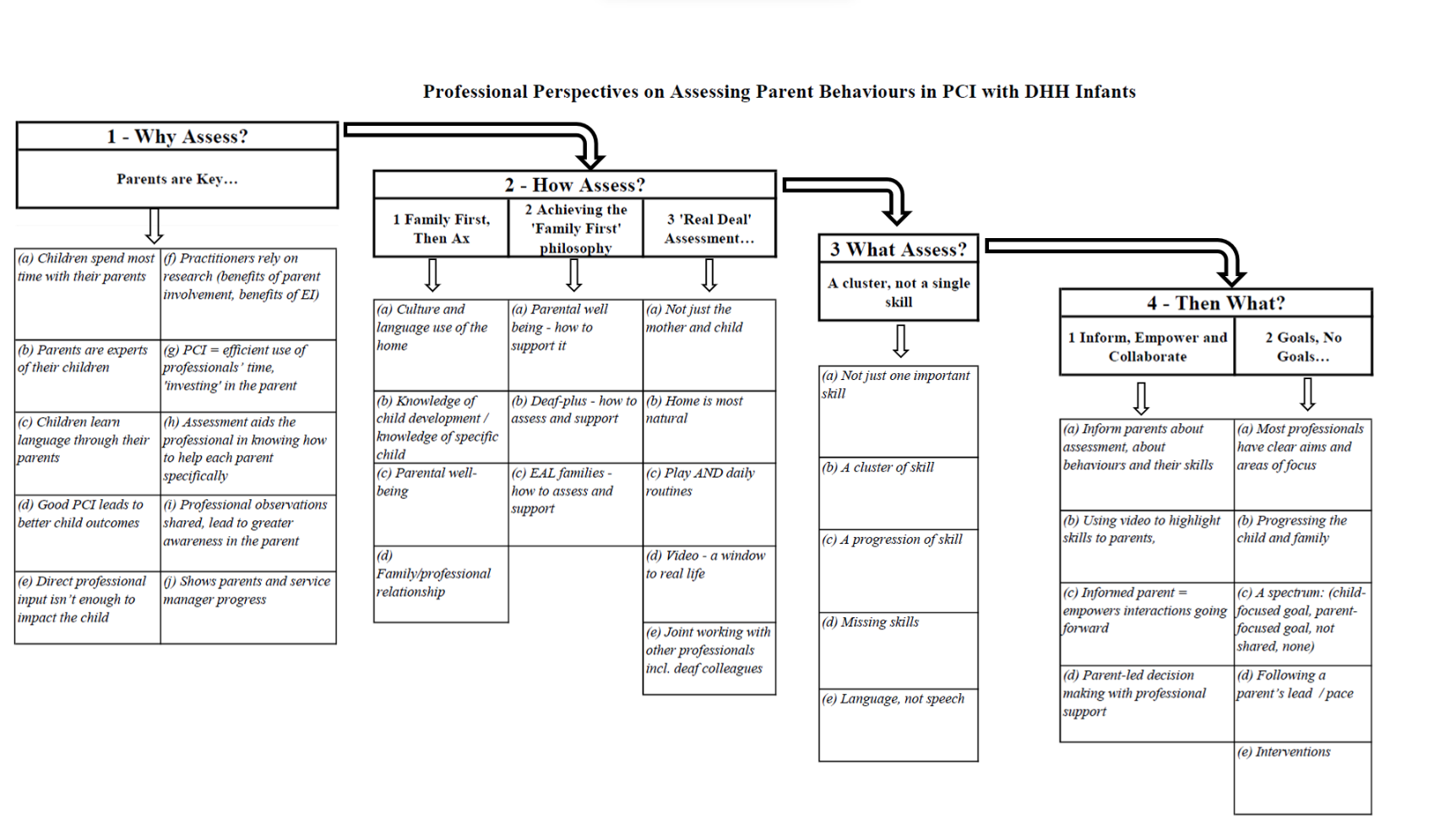
***

Supplement: Supplementary file 2 [file Table_2.DOCX]
