# Supplementary material for: “It doesn’t matter if we’re the most amazing professionals in the world…” A qualitative study of professionals’ perspectives on parent-child interaction assessment with deaf infants
Source: Front Psychol. 2024 Mar 4;15:1315220. doi: 10.3389/fpsyg.2024.1315220 (PMC10944883; doi:10.3389/fpsyg.2024.1315220)
Supplement: Supplementary file 3 [file Data_Sheet_1.DOCX]

***Appendix A – Focus Group Topic Guide***

1 Why do so many professionals assess or observe PCI?

2 Based on the top ten parent-child behaviours professionals assess (Curtin et al., 2023), is there a most important skill? Is there a cluster?

3 Which skills are fundamental to any language/modality?

4 Which behaviours are missing from the top 10?

5 Can you describe a best practice way of assessing parent-child interaction?

6 What adaptations are needed for families who use a language other than English? For families with children with additional needs?

7 In which ways do you monitor parents’ acceptance of deafness, parental well-being, self-efficacy with parenting a deaf child?

8 Describe how you decide on a goal.

9 Beyond goals, what do you do with the information from your PCI assessments?
